# An exploration of how specialist dementia nurses perceive and maintain the skills and competencies that frame their specialism: A qualitative survey

**DOI:** 10.1016/j.heliyon.2024.e27856

**Published:** 2024-03-22

**Authors:** Pat Brown, Claudia Cooper, Karen Harrison Dening, Juanita Hoe, Alexandra Burton

**Affiliations:** a Division of Psychiatry, University College London, London, UK & Dementia UK, London, UK; b Centre for Psychiatry and Mental Health, Wolfson Institute of Population Health, Queen Mary University of London, London, UK, And East London NHS Foundation Trust; c School of Health & Life Sciences, De Montfort University, Leicester, Leicestershire & Dementia UK, London, UK; d Geller Institute of Ageing and Memory (GIAM), University of West London, London, UK; e Department of Behavioural Science and Health, University College London, London, UK

## Abstract

**Background:**

UK policy for complex and long-term health conditions including dementia has recommended that specialist nursing intervention is offered across the trajectory of the condition, but there is a lack of agreement regarding the skills and competencies that specialist nurses are expected to possess. Admiral Nurses are the largest UK group of specialist dementia nurses.

**Objective:**

To explore how Admiral Nurses met and were supported to meet competencies as defined in the Admiral Nurse Competency Framework, and to develop and maintain skills as dementia specialists.

**Design:**

Cross-sectional, semi-structured survey.

**Setting:**

Online national survey.

**Participants:**

Admiral (specialist dementia) Nurses.

**Methods:**

We co-designed our survey with Admiral Nurses; then invited Admiral Nurses to complete it in 2022-23 Data were analysed thematically.

**Results:**

68 (20% of all Admiral Nurses) completed the survey; most were female (85.2%), from a white ethnic group (88.2%); they reported on average 24 years of nursing experience. We identified three themes in responses: **1.***Having time and skills for meaningful* support, explored how participants were resourced with time and skills to understand and address family carer client needs by active listening, tailoring person-centred support, and “walking alongside” families. **2.***Partnering family carers*, concerned how they co-designed interventions with family carers, learning from these collaborative partnerships where expertise was shared. **3.***Practice and peer-based learning*, explored how participants took responsibility for using available training, peer learning and self-reflection to develop their practice.

**Conclusions:**

Admiral Nurse roles enabled respondents to develop as autonomous practitioners and to access resources that supported them to build and sustain their dementia specialist practice. Learning was practice based, through partnerships with family carer clients, peer support and self-directed learning. Specialist nursing models may help address the global health workforce emergency, through enabling creative practice development and valued roles that support retention of experienced nurses.

**What is already known**

• A highly skilled workforce is needed to support families managing the complexities of dementia.
• To develop as an advanced specialism, dementia nursing needs to define the core skills and competencies of the role.
• Families value nurses who are experts in dementia with whom they can develop a supportive ongoing relationship.

**What this paper adds**

• Admiral Nurses (specialist dementia nurses) described their role as distinct in the degree of autonomy, time, and resources they have, to co-create care with family carers that is carer-centred.
• Role-specific learning built on nurses' extensive pre-existing skills, was practice based, with learning through partnerships with family carer clients, peer support and self-directed learning.
• Specialist dementia nursing may be critical to addressing the global health workforce emergency, as a model that provides opportunities for creative practice development.

## 1 Background

Dementia is common, with over 55 million people affected worldwide [1]. In the UK, over 900,000 people have been diagnosed with dementia. Many have complex needs, and over 77% have at least one co-morbid condition [2]. Family carers provide support that is instrumental to upholding the quality of life of people living with dementia [3]. Support of the person with dementia from family carers has been found to extend the time they are able to live in their own homes [4], yet the psychological impact can be high for carers [5,6] and carer-dyad relationships can be complex. Globally, it is recognised that a highly skilled workforce is needed to support families managing the complexities of dementia, yet efforts to train the health and social care workforce in dementia care core competencies, particularly regarding the assessment and treatment of carer distress, remain insufficient [7]. UK policy recommends that families who are living with dementia and have complex needs receive ‘high quality’ support and interventions [8,9].

Specialist nurse roles include Clinical Nurse Specialist, Nurse Practitioner and Advanced Nurse Practitioner, with global variation in terminology [10]. There is a shared understanding that specialist nurses require a high level of condition-specific expertise [11], and a relevant master's level education [12]. The specific skills and competencies they require in dementia care remains ambiguous [13,14]. They may, for example, offer a range of psychological interventions to support family carers and mitigate the impact of their caring role [15], improve care co-ordination for people with dementia and their family carer, and promote partnership working [8].

Admiral Nurses are the largest UK group of specialist dementia nurses, supported by the charity Dementia UK and hosted across NHS, social care, voluntary, and charity sector organisations. Admiral Nurses provide support and specialist interventions to the whole family who are affected by dementia, with a focus on supporting the family carer [16]. They support people with complex needs associated with a dementia diagnosis across healthcare settings [17].

Competency in nursing has been described as a complex combination of knowledge, skills, professional judgement, values, and attitudes which are context-specific [18]. One study has developed a competency measure for specialist nursing in Finland, however acknowledged some barriers regarding implementation, and inconsistencies in the conceptualisation of specialist nursing and competencies [12]. In the UK, Admiral Nurses have adopted the Admiral Nurse Competency Framework (ANCF) to describe and measure the attributes, specialist interventions, and essential competencies for this specialist role [19]. No previous study has explored how Admiral Nurses adopt these competencies in practice. We aimed to explore how Admiral Nurses met and were supported to meet competencies as defined in the Admiral Nurse Competency Framework and to develop and maintain skills as dementia specialists.

## 2 Methods

### 2.1 Survey design and development

The online survey was hosted using a secure online platform provided by Qualtrics [20]. It was developed in four stages. First, we conducted a systematic review [21] to identify knowledge gaps in specialist dementia nursing models. We used this, and the Admiral Nurse Competency Framework [19] to inform development of open-ended questions that captured constructs of Admiral Nursing competencies. Next, the lead researcher (PB) convened a virtual focus group of six Admiral Nurses, who worked in community settings, and on the Admiral Nurse dementia helpline, with a length of service ranging from 0 to 20 years. Attendees shared their initial thoughts on an early draft of the survey questions designed to address the survey aim. A second group of Admiral Nurses piloted the survey (n = 4), reporting on face and content validity of the questions [22,23].

The final electronic survey comprised nine questions about sociodemographic status and nursing role experience (see Table 1 and Appendix A); then 17 open-ended questions, of which three sought general perceptions about the Admiral Nurse role. The remainder asked about how the Admiral Nurses perceived they achieved and applied the specific competencies contained within the Admiral Nurse Competency Framework. Competency-based questions mirrored the six competency domains contained within the ANCF: *Person-centred care*, *Therapeutic skills, Triadic relationship, Sharing knowledge, Best practice, and Critical reflective practice* [19]. The survey was designed to identify barriers and facilitators that may influence the acquisition of knowledge, skills, or competencies.

### 2.2 Survey sample

Our target population was Admiral Nurses working in the UK (n = 339 at time of survey launch, October 1, 2022) First, PB contacted 149 nurses who had agreed to be contacted about future research during the annual census survey and thus formed a convenience sample for the study. Six additional Admiral Nurses were recruited via a work-based online learning platform (Blackboard learning) available to all Admiral Nurses as part of their practice development.

### 2.3 Procedure for distribution of the survey and data collection

PB emailed an anonymous link to prospective respondents via Qualtrics [20], an online survey software hosting platform, including a participant information sheet. Qualtrics has an in-built mechanism that prevents a duplicate survey being completed. Survey respondents are sent an email link to access the survey; this can only be completed once. Participants were able to start the survey once they had read the information sheet and indicated their consent. Emails were initially sent between October 2022 and January 2023; reminder/follow-up emails were sent between November 2022 and January 2023. Responses were held securely and anonymously within the Qualtrics software, then exported to an Excel spreadsheet for analysis. (Survey questions are available as supplementary material, Appendix A).

### 2.4 Data analysis

We used standard descriptive statistics to summarise demographic data (Table 1). Qualitative data were exported to NVivo 12™ for analysis [24]. We adopted a constructionist approach [25] that considered the meanings of the responses elicited, and the influence of the social context. A hybrid approach of deductive and inductive thematic analysis was adopted [26,27] by starting with coding and categorisation of the data [28] and a set of codes were formed which corresponded to the six domains of the Admiral Nurse Competency Framework. Keeping the hybrid approach (to thematically analysing the data) in mind [26], PB immersed themself in the data, and identified new codes, adding these to the ‘parent’ codes that were already created. Whilst the approach started with a deductive coding frame, new codes were inductively developed and added as responses were read and re-appraised. Codes that shared a common thread or had similar characteristics or features were grouped together to form initial themes which were refined and developed into final themes.

The codes and themes were discussed and iteratively reviewed with the co-authors to ensure that the data within each theme was coherent and relevant to the selected theme [28,29]. PB kept an ongoing reflexive account of the analysis process and checked her findings periodically with the co-authors as the analysis progressed [29]. Authors considered their positions, for example as Admiral Nurses (PB, KHD, JH), a psychiatrist (CC) and researchers (all) throughout the analysis process [29]. They regularly discussed the findings to ensure that the analysis remained relevant and true to the research aims and questions [30].

The study received ethical approval from University College London in August 2022 (REF.22837/001). All participants provided informed consent before commencing the survey.

## 3 Results

### 3.1 Description of the sample

Of the 339 invitations sent, 68 surveys were completed, of which 16 were only partially completed. This represents 68/339 (20%) of all potential participants. Sociodemographic and role characteristics of participants are presented in Table 1. Most participants were female (85.3%, n = 58), aged 50 and over (63.2%; n = 43), from a white ethnic group (88.2%; n = 60); and experienced in nursing (mean of 24 years of experience, six as an Admiral Nurse).

**Table 1:** Summary demographics of the survey respondents (n = 68).

Characteristics	Category	N/Mean where stated (%/Range where stated)
Age (Years)	18-29 30-39 40-49 50+	3 (4.4%) 7 (10.3%) 15 (22.1%) 43 (63.2%)
Gender	Female Male Trans-gender Non- binary Preferred not to answer Other	58 (85.3%) 9 (13.2%) 1 (1.5%) 0 0 0
Ethnicity	Black/African/Caribbean/Black- British Mixed/Multiple ethnic groups Other ethnic group White	2 (2.9%) 2 (2.9%) 2 (2.9%) 60 (88.2%)
Nursing experience (years)	Not categorised (Free text)	Mean 24.3 (Range 3.5–45)
Years as an Admiral Nurse	Not categorised (Free text)	Mean 6.1 (Range 0.5–22)
Current nursing registration (Respondents could select all that apply)	Registered Nurse – Mental Health Registered Nurse – Adult Registered Nurse – Learning Disability Other Registered Nurse	44 (64.7%) 16 (23.5%) 1 (1.5%) 6 with Dual registration (8.8%)
Qualifications	Diploma/Certificate Bachelor, Postgraduate Diploma/Certificate/Masters PhD Did not state/complete	22 (32.4%) 34 (50%) 6 (8.8%) 6 (8.8%)

### 3.2 Qualitative analysis findings

We identified three themes. The first theme, *having time and skills for meaningful* *support*, explored how participants were resourced with time and skills to get to know, support and understand the needs of family carer clients. Subthemes described how they spent time: to truly listen (subtheme 1), to tailor person-centred support matched to need (subtheme two) and to “walk alongside" the family carer; in this practice they developed and maintained competencies in compassionate care (subtheme three). The second theme, *partnering family carers*, concerned how participants worked with family carers to co-design bespoke interventions, learning from these collaborative partnerships in which expertise was shared. The third theme**,** *practice and peer-based learning*, explored how participants used training, peer learning and self-reflection to develop their practice and were supported in this, or not, by organisational structures such as protected time and timetabled learning. Themes and subthemes are described in more detail in Table 2.

**Table 2:** Themes and subthemes.

THEME	SUBTHEME	EXCERPTS
Theme 1., Having time and skills for meaningful support	• The skilled and holistic listener • Matching intervention to needs, not a “Blanket approach as everyone is different”. • ‘Walking alongside’, focusing also on the family carer	*‘Listening and taking individual interests, needs, wants into consideration. Recognising the individuality in all, and how their lives will impact on them and how they cope with dementia/live with dementia’. (Female, 25–30 years' nursing experience, and between 5-10 years as an Admiral Nurse)* *‘I do not use a blanket approach, as everyone is different however, I try and offer a solution focused approach to help with practical issues families have’.(Female, 25–30 years' nursing experience, and between 5-10 years as an Admiral Nurse)* *‘ … there is more time, more focus on the triadic nature of relationships and the impact of conditions (dementia and other) on everyone involved’. (Female, between 5-10 Years' nursing experience)*
Theme 2., Partnering family carers		*‘Carer-led’ (Female, 25–30 years' nursing experience, and under 5 years as an Admiral Nurse)* *‘Carers are the focus, and their voice is heard now which enables me to ensure they stay as well as possible, emotionally and physically throughout their caring role experience.’ (Female, over 35 years' nursing experience, and 15 years as an Admiral Nurse)* *‘Listening to what people say they need. Hearing their story. Looking at what is important to them and ensuring they are involved in designing their own care plan wherever possible or involving significant others who can advocate for them.’ (Female, 30–35 years nursing experience, and 5–10 years as an Admiral Nurse)*
Theme 3., Practice, and peer- based learning		*‘Mainly learning on the job through the Practice Action Learning sessions, Learning from other admiral nurses. (Female, 20–25 years’ nursing experience, and under 5 years as an Admiral Nurse)* ‘*The information provided in the form of courses, short learning units and webinars available on Blackboard are all based on the most up to date information, which means I have access to this at all times.‘(Female, 20–25 years nursing experience and 5–10 years as an Admiral Nurse)* *‘This is largely self-driven/managed.’ (Female, 40–44 years nursing experience and over 15 years as an Admiral Nurse)*

#### 3.2.1 Theme 1

The time and skills to offer meaningful support.

This theme explored how participants provided compassionate case management, with a depth of engagement possible through advanced listening and assessment skills (subtheme 1), the flexibility to work in partnership to tailor their approach to client needs (subtheme 2), and the focus on family carers (subtheme 3). This was at times contrasted to traditional multidisciplinary models, where the greatest hours of client-facing time are focussed on the person with dementia rather than the family carer, and intervention planning is multidisciplinary team or medical-led, rather than nurse-led.

##### 3.2.1.1 Subtheme 1: the skilled and holistic listener

Numerous participants described using listening and communication skills to ensure the client was heard:

*‘I think families feel listened to. I think they feel that we truly understand what they are going through, and I think they are grateful for the knowledge we have and can share with them to help them understand what is happening to them.’ (Female, with over 25 years nursing experience, 5–10 years as an Admiral Nurse)*

Listening skills were often described as critical to enabling the needs of all members of the family who were affected by dementia to be assessed, and for appropriate interventions to be employed:

*‘I listen attentively in person and on the phone, I aim to address individual need, this may be by signposting to another professional/organisation* … *try a day centre for the first time if carer struggling to access or taking a carer to try a group for the first time if nervous*.' *(Female, with 5-10 years’ nursing experience, under 5 years as an Admiral Nurse)*

*‘Listening and taking individual interests, needs, wants into consideration. Recognising the individuality in all and how their lives will impact on them and how they cope with dementia/live with dementia.’ (Female, with 25–30 years' nursing experience, 5–10 years as an Admiral Nurse)*

Often, the flexibility of having more time was perceived as a key driver of better, higher quality care:

*‘The Admiral Nurse role allows me to offer time to families I talk to. I am not restricted by number of contacts to be made a day or an expectation to offer quick input and then discharge. This enables depth and exploration to occur, often resulting in getting to the route of problems.’ (Female, with over 25 years nursing experience, 5–10 years as an Admiral Nurse)*

*‘I would say it is only different by the amount of time and focus I can have with families. In previous roles the time has been limited due to service pressure which reduces the quality of my support in my opinion.’ (Female, with over 5 years' nursing experience, under 5 years as an Admiral Nurse)*

That time enabled a depth and meaningfulness of engagement was also shown through the impact on Admiral Nurses. The following quote alludes to the depth of emotional labour need to provide support that was, at times intensive:

*‘The time spent with the family tends to be for much longer than in my previous roles and requires a greater degree of emotional and psychological support.’ (Female, with over 20 year’s nursing experience, 10–15 years as an Admiral Nurse)*

##### 3.2.1.2 Subtheme 2: matching intervention to needs: not *“a blanket approach, as everyone is different”*

Most participants described the breadth of interventions that they used to respond to the needs of the family carer, or whole family, both directly and by acting as a conduit between ‘services’ (Statutory and non-statutory), and the family:

*‘ … being able to contact a GP on someone's behalf, arranging food parcels or other supplies, getting other needed services on board.’ (Female, with 5–10 years nursing experience, under 5 years as an Admiral Nurse)*

Sometimes Admiral Nurses described specific interventions or selected from a ‘toolkit’ of approaches:

*‘I do not use a blanket approach, as everyone is different however, I try and offer a solution focused approach to help with practical issues families have … I also find encouraging use of music, relaxation and other anxiety reducing methods are very useful.’ (Female, with over 25 years nursing experience, 5–10 years as an Admiral Nurse)*

*‘Many of my own approaches utilise psychological interventions based on counselling to create a therapeutic environment… using different assessment tools like Becks Cognitive Triad can utilise CBT interventions where 'we' have been able to explore this cognitive perspective.’ (Female, with over 30 years nursing experience, 20–25 years as an Admiral Nurse)*

Several nurses described how a depth of skills may be enabled by the focus on dementia, in contrast to the breadth of expertise required in more generic roles:

*‘ … it enables to be completely specific to the needs of people with dementia, other roles previously, priority was given to other mental health conditions.’ (Female, with over 20 years nursing experience, under 3 years as an Admiral Nurse)*

*‘Requirement to have experience in dementia care and training / qualification prior to coming into post. Focus on offering psychosocial support to the whole family - as opposed to just the person with dementia.’ (Female, with over 30 years nursing experience, 20–25 years as an Admiral Nurse)*

##### 3.2.1.3 Subtheme 3: ‘walking alongside’: focusing also on the family carer

Many nurses in the previous examples highlighted that one of the key differences between their specialist role and previous nursing role(s) was the relational aspect of working with the whole family, to include family carers. In the next excerpt, the nurse compares her practice as an Admiral Nurse to how they worked previously:

*‘Providing individualized(sic) support and therapeutic interventions through the development of a supportive relationship …. Previously wouldn't have assessed carers needs and/or offered individual support/ counselling / interventions.’ (Female, with over 30 years nursing experience, 20–25 years as an Admiral Nurse)*

In the following example, the Admiral Nurse describes how they focussed on hearing the family carer's ‘story’ (often told for the first time):

*‘Family often express that an admiral nurse is the first person to listen to family concerns rather than just focusing on the 'patient'.’ (Female, with 15–20 years nursing experience, 10–15 years as an Admiral Nurse)*

Another participant contrasts the orientation of Admiral Nursing services to ‘family carer-focussed’ from ‘patient-focussed’ nursing roles:

*‘The focus on supporting carers is fundamentally different and unusual compared to essentially all other nursing roles in dementia … ensuring that the person's voice and wishes are heard, listened to, and understood and acted upon wherever possible, no matter how difficult this may sometimes be.’ (Female, with 5–10 years nursing experience, under 1 year as an Admiral Nurse)*

The depth of work with family carers was likened by one respondent as “walking alongside”:

*‘ … this process of guidance and ‘walking alongside’ long term through the array of symptoms, emotions, dilemmas, service negotiation and advocacy. No other service remains involved with a PWD and their family long-term.’ (Female, with over 35 years nursing experience, under 5 years as an Admiral Nurse)*

Admiral Nurses described the perceived individual and family benefits and impact of their intervention:

*‘ … the positive impact it has made along with statements on how it has reduced carer stress and potential carer breakdown. The feedback also shows that the support of the service has prevented people from accessing emergency services of the GP.’ (Female, with 20–25 years nursing experience, 5–10 years as an Admiral Nurse)*

*‘Families not being fearful of the future … understanding of systems and processes that can help them as well as the person living with dementia.’ (Female, with over 35 years nursing experience, 5–10 years as an Admiral Nurse)*

*‘The realisation that they do not have to do this alone, ….is so empowering, liberating and gives a sense of burdens being shared just a little.’ (Female, with 30–35 years nursing experience, under 3 years as an Admiral Nurse)*

While there was overwhelming support for this family carer-centred model, one respondent described their experience of trying to balance the needs of both parties in the care-dyad:

‘*Can be hard’ (Female, with 25-30 years nursing experience, 5–10 years as an Admiral Nurse)*

#### 3.2.2 Theme 2: Partnering family carers

This theme concerned how Admiral Nurse participants worked with family carers to co-design bespoke interventions, learning from these collaborative partnerships in which expertise was shared:

*‘ … listen to the needs of the families/carers they support to identify how to offer the right support for them.’ (Female, with 30–35 years nursing experience, 5–10 years as an Admiral nurse)*

*‘You listen to what they need … the family drive the process.’ (Female, with 35–40 years' nursing experience, 5–10 years as an Admiral Nurse)*

*‘A lot more time is given to really getting to know the carer, hearing their story, digging deep to pick up all the hidden issues that are so influential to how they are coping.’ (Female, with 30–35 years nursing experience, under 5 years as an Admiral Nurse)*

*‘We are able to build rapport, gain trust, afforded a link into their family/carer life, to enable them to become empowered, the PWD expert.’ (Female, with 25–30 years nursing experience, under 5 years as an Admiral Nurse)*

#### 3.2.3 Theme 3: ‘Practice and peer-based learning’

Theme three explores how participants used training, peer learning and self-reflection to develop their practice and were supported in this, or not, by organisational structures such as protected time and timetabled learning.

As might be expected, given that respondents had on average 24 years of nursing experience, many reported entering their Admiral Nurse role already equipped with skills and extensive subject-area knowledge:

*‘I have learnt these ways of working over many years of nursing.’ (Female, with 25–30 years nursing experience, 5–10 years as an Admiral Nurse)*

*‘Initially acquired skills through previous roles, degree training + CPD around therapeutic approaches. Supported by CPD through host / Dementia UK to build on these skills.’ (Female, with 5–10 years nursing experience, under 5 years as an Admiral Nurse)*

Proactive and self-directed learning styles were most commonly described:

*‘I am proactive with continued professional development and spend a lot of time reading to keep up to date with current evidence base and develop knowledge in areas I am less familiar with.’ (Female, with 15–20 years' nursing experience, under 5 years as an Admiral Nurse)*

*‘(Learning) … This is largely self-driven/managed.’ (Female, with over 40 years nursing experience, 15–20 years as an Admiral Nurse)*

*‘ … utilising up to date research sourced myself or that is provided by the charity via the research and publications teams and the practice development team.’ (Male, with 25–30 years nursing experience, 15–20 years as an Admiral Nurse)’*

This self-direction sat alongside provision of considerable structured resources:

*‘Through experience of the role, listening to fellow Admiral Nurses and the learning and development provided by Dementia UK … We attend webinars, sessions on Blackboard [online learning platform] and have an annual forum where we can share ideas and get up to date information. Monthly supervision is also provided which lets me discuss any cases I have finding difficult … this all enables me to feel I am working with up-to-date best evidence.’ (Female, with 25–30 years nursing experience, 5–10 years as an Admiral Nurse)*

*‘Attended training courses Completed Masters in advanced dementia, reading / learning attending webinars / learning events Information from / liaison with other professionals Supported by employer, Dementia UK*.*’ (Female, with 30-35 years nursing experience, 20–25 years as an Admiral Nurse)*

The value of peer-support, offered through ‘Practice Action Learning sets’ (PALS) peer supervision groups, was frequently referenced as a mechanism to share practice cases, reflect, and acquire group peer support:

*‘Dementia UK continually provides various mediums in the form of courses and webinars that encourage us to share good practice. These are juxtaposed with practice action learning teams that encourage us to reflect and discuss current cases and again share good practice.’ (Female, with 30–35 years of nursing experience, under 5 years as an Admiral Nurse)*

*‘I speak up in our PAL's (practice associated learning) sessions with fellow Admiral nurses and I value their feedback.’ (Female, with 5–10 years nursing experience, under 5 years' as an Admiral Nurse)*

‘*Attending PALS sessions in person helped me gain knowledge of how other Admiral Nurses worked and enabled me to learn about other sources of support for families. Practice development sessions also broadened my knowledge and helped me change my own focus from the PWD to the needs of the family carer.’ (Female, with 20-25 years nursing experience, 5–10 years as an Admiral Nurse)*

There were contrasting accounts of how the time for learning was resourced. In the next example the Admiral Nurse explained that a lot of their learning needs were accommodated in their own time:

*‘Do a lot of own learning in own time out of interest to further knowledge and apply learning to practice.’ (Female, with over 30 years nursing experience, 10–15 years' as an Admiral Nurse)*

Similarly, another nurse shared that finding time to access all the available training could be difficult:

*‘The Admiral Nurse Academy has a lot of training available for access. However, when the demands of day-to-day work are so great it takes a lot of willpower to set aside the time to access this, even when you know it will enhance practice.’ (Female, with over 30 years nursing experience, under 5 years as an Admiral Nurse)*

This was contrary to the accounts of many nurses who reported having learning-specific time within their working week:

*‘We have a clinical effectiveness day weekly where someone presents a case, new research, a therapeutic approach, or service evaluation. other study days also held for staff as a planned approach or ad hoc training if required.’ (Female, with 30–35 year’s nursing experience, under 5 years as an Admiral Nurse)*

*‘I am very fortunate as my organisation allows the time for me to complete regular contact through my Pals (Practice Action Learning sets) group with Dementia UK who also provide regular training via blackboard and other courses such as the bereavement and loss sessions.’ (Female, with 20–25 years nursing experience and 5–10 years as an Admiral Nurse)*

## 4 Discussion

Respondents described how their specialist role was distinct from other nursing roles they had undertaken in focusing on the family carer as the primary client, the acquisition and utilisation of dementia and family/relationship-specific skills, knowledge, and competencies for practice. Nurses described how self-directed, practice-based learning supported them to draw creatively and autonomously on these competencies.

We noted some symmetry between what respondents provided to clients, time to listen, advanced skills and access to resources, and how their own development needs were met – through having time and resources to train and reflect with peers. Other studies have also described how the quality of health care [31,32] and nurses’ continuing professional development are adversely affected when there is a lack of time and support to engage in nursing roles [33].

The Admiral Nursing model of dementia care explicitly acknowledges the carer's interconnectedness with the person living with dementia, and their centrality to facilitating healthcare delivery [34]. Respondents viewed their relationship with the family carer as valuable and as partnerships to facilitate holistic assessment and needs-led interventions. This focus on building a ‘meaningful relationship’ with family carers has been previously cited as highly valued by clients [17].

For the nurses, the access to, and provision of resources enabled their practice as autonomous, confident nurse-specialists, attributes that family carers value [17]. Some respondents described pre-existing expertise, and many described how they were actively supported to augment their skills, via a programme of ongoing practice development. To practice at an advanced or specialist level, nurses are required to actively reflect on their clinical practice [35,36]. Within Admiral Nursing, reflective practice is enabled through Practice Action Learning Sets (PALS); time-protected and mandated within the Admiral Nurses’ practice development programme [37]. Several respondents cited Practice Action Learning sets as a forum for reflection and sharing good practice. New knowledge, skills acquisition, and competencies identified within the Admiral Nurse Competency Framework [19] were acquired from a raft of training and education opportunities available.

The survey responses illustrated situations where the Admiral Nurse had identified, prioritised, and autonomously directed care and support to meet the needs of the individuals within the family context. Many respondents articulated their abilities and maturity in managing complex, relationship challenges [38].

The long duration of nursing service of most respondents reflects the relative seniority and experience of specialist nurses, and competence, problem-solving ability, and confidence, acquired through this experience [39]. Most respondents were older, more experienced nurses, a population in whom workforce retention is particularly challenging [40].

Our findings add to evidence supporting the implementation of specialist nurses for families affected by dementia [8,21]. We have highlighted key skills, and competences that specialist nurses who work with this client group perceive are of benefit to them in their role. Our findings accord with a recent review of enablers of effective Continuing Professional Development; these included autonomy and motivation of learners, linking topics of learning to practice, and a supportive and enabling workplace [41].

The provision of specialist dementia nursing has been found to be no more costly than traditional, non-dementia-specific models, and family carers valued the ongoing support and relationship with a professional who was expert in their knowledge about dementia [17]. These findings, and those from a recent systematic review of specialist dementia nursing models [21] suggest there is scope to develop more cost-effective specialist nursing and nurse-led models for dementia. These may be useful in considering how to meet global workforce needs, including in Low- and Middle-Income countries (LMIC) where nurse-led services may support solutions for resourcing a dwindling nursing workforce amid a global health workforce challenge where the World Health Organizationbelieves that there will be a worldwide shortfall of 18 million health workers by 2030 [42].

## 5 Limitations

Our response rate (20% of the total workforce) indicates that we cannot claim our findings are necessarily representative of all Admiral Nurses, although in terms of gender balance, the proportion of female respondents was broadly representative of all Admiral Nurses, as found in their 2022 census (85% versus 89% in census); and in the proportions from census and survey populations from a white ethnic group (87% for census, 88.2% for the survey) (Dementia UK personal communication). As we recruited via a learning platform, nurses who were more engaged with Continuing Professional Development (CPD) may have been more likely to participate. PB, KHD and JH are Admiral Nurses. PB attempted to mitigate potential for confirmation bias through active reflection, and team discussion involving all co-authors including CC and AB who are not Admiral Nurses.

## 6 Conclusions

Admiral Nurse roles are distinct in the degree of autonomy, time and resources that support nurses to sustain and develop their dementia specialist practice. Learning is often built on extensive pre-existing skills and is practice based through partnerships with family carer clients, peer support and self-directed learning. Specialist nursing has previously been found to be as cost-effective as traditional multidisciplinary models. Our findings suggest they may be critical to addressing the global health workforce emergency, as a model that provides opportunities for creative practice development.

## Data availability statement

The study data will be made available on request and has not been deposited into a publicly available repository.

## CRediT authorship contribution statement

**Pat Brown:** Writing – review & editing, Writing – original draft, Visualization, Methodology, Formal analysis, Data curation, Conceptualization. **Claudia Cooper:** Writing – review & editing, Supervision, Methodology, Formal analysis, Conceptualization. **Karen Harrison Dening:** Writing – review & editing, Supervision, Methodology, Conceptualization. **Juanita Hoe:** Writing – review & editing, Supervision, Conceptualization. **Alexandra Burton:** Writing – review & editing, Supervision, Methodology, Formal analysis, Conceptualization.

## Declaration of competing interest

The authors declare that they have no known competing financial interests or personal relationships that could have appeared to influence the work reported in this paper.
