# Supplementary material for: An exploration of how specialist dementia nurses perceive and maintain the skills and competencies that frame their specialism: A qualitative survey
Source: Heliyon. 2024 Mar 22;10(7):e27856. doi: 10.1016/j.heliyon.2024.e27856 (PMC11001773; doi:10.1016/j.heliyon.2024.e27856)
Supplement: Multimedia component 1 [file mmc1.docx]

**Checklist for Reporting Of Survey Studies (CROSS)**

| **Section/topic** | **Item** | **Item description** | **Reported on page #** |
| --- | --- | --- | --- |
| **Title and abstract** | | |  |
| Title and abstract | 1a | State the word “survey” along with a commonly used term in title or abstract to introduce the study’s design. | √  This is included on the Title page (Separate document) and on the header of the manuscript |
|  | 1b | Provide an informative summary in the abstract, covering background, objectives, methods, findings/results, interpretation/discussion, and conclusions. | Page 2 of the Title page includes the abstract which covers all required sections (Background, objective etc. ‘Discussion’ has been integrated into ‘Results’ & ‘Conclusions’) |
| **Introduction** | | |  |
| Background | 2 | Provide a background about the rationale of study, what has been previously done, and why this survey is needed. | A summary (Bullet- point) of what is already known is provided on page 1 of the ‘without author’ manuscript.  A full background is on page 2 with last paragraph on page 2 providing the rationale for undertaking the study. |
| Purpose/aim | 3 | Identify specific purposes, aims, goals, or objectives of the study. | Aim of the study (Survey) is captured in the last paragraph on page 2 of the ‘without author’ manuscript. |
| **Methods** | | |  |
| Study design | 4 | Specify the study design in the methods section with a commonly used term (e.g., cross-sectional or longitudinal). | The study design, development, and process for undertaking the survey/study is outlined on pages 3 and 4 of the ‘without author’ manuscript. |
|  | 5a | Describe the questionnaire (e.g., number of sections, number of questions, number and names of instruments used). | Please refer to supplementary material (Appendix A) and also page 3 paragraph 2. |
| Data collection methods | 5b | Describe all questionnaire instruments that were used in the survey to measure particular concepts. Report target population, reported validity and reliability information, scoring/classification procedure, and reference links (if any). | The survey was developed initially from the identification of knowledge gaps identified within a recent systematic review (Page 3, para. 1) and a desire to ask more questions arising from the systematic review findings. The survey questions were framed around the Admiral Nurse competency Framework referred to on page 3, paragraph 1) The target population were Admiral Nurses, detailed under the heading ‘Survey sample’ on page 3, paragraph 3. Pre- testing is described in the first paragraph on page 3 of the manuscript (See also below) |
|  | 5c | Provide information on pretesting of the questionnaire, if performed (in the article or in an online supplement). Report the method of pretesting, number of times questionnaire was pre-tested, number and demographics of participants used for pretesting, and the level of similarity of demographics between pre-testing participants and sample population. | Testing/piloting of the questionnaire /survey is described on page 3 of the manuscript, paragraph 1 |
|  | 5d | Questionnaire if possible, should be fully provided (in the article, or as appendices or as an online supplement). | The questionnaire has been included as supplementary information (Appendix A) |
| Sample characteristics | 6a | Describe the study population (i.e., background, locations, eligibility criteria for participant inclusion in survey, exclusion criteria). | This is described on page 3, paragraph 3 of the manuscript |
|  | 6b | Describe the sampling techniques used (e.g., single stage or multistage sampling, simple random sampling, stratified sampling, cluster sampling, convenience sampling). Specify the locations of sample participants whenever clustered sampling was applied. | A convenience sample was used which is described in paragraph 3 on page 3 of the manuscript |
|  | 6c | Provide information on sample size, along with details of sample size calculation. | Sample size is described in paragraph 3, page 3 of the manuscript |
|  | 6d | Describe how representative the sample is of the study population (or target population if possible), particularly for population-based surveys. | Representativeness of the sample is discussed under ‘limitations’ on page 15 of the manuscript |
| Survey  administration | 7a | Provide information on modes of questionnaire administration, including the type and number of contacts, the location where the survey was conducted (e.g., outpatient room or by use of online tools, such as SurveyMonkey). | This information is found in paragraph 4, page 3 under the heading ‘Procedure for distribution …’ |
|  | 7b | Provide information of survey’s time frame, such as periods of recruitment, exposure, and follow-up days. | This is described in paragraph 4, page 3 of the manuscript |
|  | 7c | Provide information on the entry process:  –>For non-web-based surveys, provide approaches to minimize human error in data entry.  –>For web-based surveys, provide approaches to prevent “multiple participation” of participants. | The procedure for entering the survey software to complete the survey is described on page 3, paragraph 4. |
| Study preparation | 8 | Describe any preparation process before conducting the survey (e.g., interviewers’ training process, advertising the survey). | The survey was designed and prepared with the involvement of a focus group of nurses and piloted by a different group of nurses. Details are provided on page 3 first paragraph. Advertising for recruitment purposes is described on page 3, paragraph 3. |
| Ethical considerations | 9a | Provide information on ethical approval for the survey if obtained, including informed consent, institutional review board [IRB] approval, Helsinki declaration, and good clinical practice [GCP] declaration (as appropriate). | Ethical approval is mentioned on page 4, second paragraph |
|  | 9b | Provide information about survey anonymity and confidentiality and describe what mechanisms were used to protect unauthorized access. | The ‘Qualtrics’ survey platform has an in-built anonymous link that was created and sent to prospective respondents : <https://www.qualtrics.com/support/survey-platform/distributions-module/web-distribution/anonymous-link/>. The link is mentioned on page 3 of the manuscript under heading ‘Procedure …’ |
| Statistical  analysis | 10a | Describe statistical methods and analytical approach. Report the statistical software that was used for data analysis. | Data analysis is described on page 3 |
|  | 10b | Report any modification of variables used in the analysis, along with reference (if available). | Not applicable (qualitative survey) |
|  | 10c | Report details about how missing data was handled. Include rate of missing items, missing data mechanism (i.e., missing completely at random [MCAR], missing at random [MAR] or missing not at random [MNAR]) and methods used to deal with missing data (e.g., multiple imputation). | . Number of incomplete surveys is given on page 4 under ‘Results’ |
|  | 10d | State how non-response error was addressed. | This is discussed under ‘Results’ page 4, paragraph 3. |
|  | 10e | For longitudinal surveys, state how loss to follow-up was addressed. | Not applicable (one off qualitative survey) |
|  | 10f | Indicate whether any methods such as weighting of items or propensity scores have been used to adjust for non-representativeness of the sample. | Not applicable (qualitative survey) |
|  | 10g | Describe any sensitivity analysis conducted. | Not applicable (qualitative survey) |
| **Results** | | |  |
| Respondent characteristics | 11a | Report numbers of individuals at each stage of the study. Consider using a flow diagram, if possible. | The number of people wo were approached is found on page 4, paragraph 3. 69 nurses started the survey, 68 completed it; although 16, whilst they finished the survey, did not answer all the questions, |
|  | 11b | Provide reasons for non-participation at each stage, if possible. | This was not possible due to participant anonymity |
|  | 11c | Report response rate, present the definition of response rate or the formula used to calculate response rate. | The response rate (20%) is stated on page 15 (under ‘limitations’ |
|  | 11d | Provide information to define how unique visitors are determined. Report number of unique visitors along with relevant proportions (e.g., view proportion, participation proportion, completion proportion). | The number of unique participants and completion proportion are described on page 4. |
| Descriptive  results | 12 | Provide characteristics of study participants, as well as information on potential confounders and assessed outcomes. | Study characteristics are detailed in Table 1, Page 5. |
| Main findings | 13a | Give unadjusted estimates and, if applicable, confounder-adjusted estimates along with 95% confidence intervals and p-values. | Not applicable (qualitative survey) |
|  | 13b | For multivariable analysis, provide information on the model building process, model fit statistics, and model assumptions (as appropriate). | Not applicable (qualitative survey) |
|  | 13c | Provide details about any sensitivity analysis performed. If there are considerable amount of missing data, report sensitivity analyses comparing the results of complete cases with that of the imputed dataset (if possible). | Not applicable (qualitative survey) |
| **Discussion** | | |  |
| Limitations | 14 | Discuss the limitations of the study, considering sources of potential biases and imprecisions, such as non-representativeness of sample, study design, important uncontrolled confounders. | Limitations are discussed on page 15-16 |
| Interpretations | 15 | Give a cautious overall interpretation of results, based on potential biases and imprecisions and suggest areas for future research. | This is covered under ‘discussion’ section (Page 14) and also within the conclusion on page 16 |
| Generalizability | 16 | Discuss the external validity of the results. | This is discussed on page 16 under the heading ‘Limitations’ |
| **Other sections** | | |  |
| Role of funding source | 17 | State whether any funding organization has had any roles in the survey’s design, implementation, and analysis. | This information can be found on page 17 paragraph 4. Under the heading, ‘funding sources’. |
| Conflict of interest | 18 | Declare any potential conflict of interest. | This is declared on page 16 |
| Acknowledgements | 19 | Provide names of organizations/persons that are acknowledged along with their contribution to the research. | All contributors to the research are stated on the Credit Author statement (Separate document) |
