# Supplementary material for: An exploration of how specialist dementia nurses perceive and maintain the skills and competencies that frame their specialism: A qualitative survey
Source: Heliyon. 2024 Mar 22;10(7):e27856. doi: 10.1016/j.heliyon.2024.e27856 (PMC11001773; doi:10.1016/j.heliyon.2024.e27856)
Supplement: Multimedia component 2 [file mmc2.docx]

**APPENDIX A**

**Supplementary material: The Admiral Nurse survey**

**DEMOGRAPHIC QUESTIONS**

**Can you indicate your age range please?**

- Under 30 🞎
- 30-39 🞎
- 40-49 🞎
- 50+ 🞎

**What gender do you identify as?**

- Male 🞎
- Female 🞎
- Trans-gender 🞎
- Non- binary 🞎
- Prefer not to answer 🞎
- Other 🞎

**What is your ethnic background? (Choose from one option that best describes your ethnic group or background)**.

- White/Caucasian 🞎
- Asian/Asian British 🞎
- Black/African/Caribbean/Black British 🞎
- Mixed/Multiple ethnic groups 🞎
- Other ethnic group (Please state) 🞎

**What is your current nursing registration? (If you have multiple registrations, please select all that apply to you)**

- Registered Nurse – Mental Health 🞎
- Registered Nurse – Adult 🞎
- Registered Nurse – Learning Disability 🞎
- Other Registered Nurse- (please state)… 🞎…………………………………………

**How long have you been a registered nurse for?** *[In years and months; Total length of time as a registered nurse]*

Years ………….. Months ………..…

**How long have you worked in an Admiral Nursing role** *[In years and months, Total length of time working as an Admiral Nurse]*

Years ………... Months ………….

**Are your working hours**

- Full- time 🞎
- Part- time 🞎

**Please select any qualifications you have from the list below**

- Diploma/Certificate
- Bachelor, Postgraduate Diploma/Postgraduate Certificate Masters
- PhD
- Other – please state……………………………………………………………………………………………….....

**Before becoming an Admiral Nurse, what nursing roles did you hold (please indicate if these roles involved working with people with dementia)?**

**Admiral Nurses are called *'Specialist dementia nurses'*. I am interested in how the way you provide care as an Admiral nurse, and are supported to do so, compares to your experiences in other nursing roles, especially when you have worked with people living with dementia.**

**PERSON CENTERED CARE**

- **How, in your experience, is the way you approach providing person-centred care similar or different to in previous roles?**
- **Thinking about any positive differences you describe; how did you learn or acquire this different way of working? How are you supported to maintain it? What impact do you think it has for clients?**
- **Is there anything about the way person-centred care is approached in Admiral nursing that you feel was better in a previous role? If so, please tell me about that**

**THERAPEUTIC SKILLS**

- **Do you think there is a way that you work people with dementia and their family that is unique to your Admiral Nurse Role, if so, please can you tell me more about the therapeutic benefit this approach affords?**
- **How have you acquired and maintained your therapeutic skills, who provided or supported you with this process**

**TRIADIC RELATIONSHIPS**

- **In your therapeutic relationships when working with families, how do you manage to address or balance the needs of everyone involved, who or what supports you to do this?**
- **Does the way you work with families share any similarities or differ to how you may have worked with families in a previous nursing role, if so, how?**

**SHARING KNOWLEDGE**

- **We asked your earlier about how you acquired and maintained your skills to practice in your role, is there a process (Formally or informally) by which you share what you have learned in practice? If so, please explain how you share the knowledge you have in this role?**

**BEST PRACTICE**

- **What, mechanisms if any, are in place to enhance your knowledge and skills when you are working with families affected by dementia, or which enables you to deliver interventions that reflect ‘Best Practice’ approaches?**

**CRITICAL REFLECTIVE PRACTICE**

- **Are there any formal or informal arrangements in place that enable you to reflect on your work and practice, if so please explain.**
- **How does this compare to the arrangements to reflect on your practice that may have been in place in previous nursing roles?**
- **Do you have any additional comments in relation to this survey?**
